# A Novel Humanized PD-1/PD-L1 Mouse Model Permits Direct Comparison of Antitumor Immunity Generated by Food and Drug Administration–Approved PD-1 and PD-L1 Inhibitors

**DOI:** 10.4049/immunohorizons.2200054

**Published:** 2023-01-19

**Authors:** Whitney Barham, Michelle Hsu, Xin Liu, Susan M. Harrington, Jacob B. Hirdler, Joanina K. Gicobi, Xingxing Zhu, Hu Zeng, Kevin D. Pavelko, Yiyi Yan, Aaron S. Mansfield, Haidong Dong

**Affiliations:** * Department of Immunology, Mayo Clinic College of Medicine and Science, Rochester, MN;; † Department of Urology, Mayo Clinic, Rochester, MN;; ‡ Division of Rheumatology, Department of Medicine, Mayo Clinic, Rochester, MN; and; § Division of Medical Oncology, Department of Medicine, Mayo Clinic, Rochester, MN

## Abstract

Seven different anti–PD-1 and PD-L1 mAbs are now widely used in the United States to treat a variety of cancer types, but no clinical trials have compared them directly. Furthermore, because many of these Abs do not cross-react between mouse and human proteins, no preclinical models exist in which to consider these types of questions. Thus, we produced humanized PD-1 and PD-L1 mice in which the extracellular domains of both mouse PD-1 and PD-L1 were replaced with the corresponding human sequences. Using this new model, we sought to compare the strength of the immune response generated by Food and Drug Administration–approved Abs. To do this, we performed an in vivo T cell priming assay in which anti–PD-1/L1 therapies were given at the time of T cell priming against surrogate tumor Ag (OVA), followed by subsequent B16-OVA tumor challenge. Surprisingly, both control and Ab-treated mice formed an equally robust OVA-specific T cell response at the time of priming. Despite this, anti–PD-1/L1-treated mice exhibited significantly better tumor rejection versus controls, with avelumab generating the best protection. To determine what could be mediating this, we identified the increased production of CX3CR1^+^PD-1^+^CD8^+^ cytotoxic T cells in the avelumab-treated mice, the same phenotype of effector T cells known to increase in clinical responders to PD-1/L1 therapy. Thus, our model permits the direct comparison of Food and Drug Administration–approved anti–PD-1/L1 mAbs and further correlates successful tumor rejection with the level of CX3CR1^+^PD-1^+^CD8 ^+^ T cells, making this model a critical tool for optimizing and better utilizing anti–PD-1/L1 therapeutics.

## Introduction

Immune checkpoint blockade (ICB) therapy has transformed the landscape of cancer treatment in the past decade. Due to a relatively manageable toxicity profile and the potential for exceptional results over a long duration, ICB has become the standard of care across many solid tumor types. After initial approvals in 2011 (anti–CTLA-4) and 2014 (anti–PD-1/L1), the number of available options for treatment has continued to increase, particularly therapies targeting PD-1/PD-L1 signaling. There are now four anti–PD-1 Abs (nivolumab, pembrolizumab, cemiplimab, and dostarlimab) and three anti–PD-L1 Abs (avelumab, atezolizumab, and durvalumab) approved in the United States for indications that stretch across at least 15 different tumor types (1, 2).

Food and Drug Administration (FDA) approval is based on clinical trials comparing each therapeutic to the standard of care, which is often chemotherapy. However, anti–PD-1/L1 Abs are never directly compared with one another. In some instances, multiple anti–PD-1 and anti–PD-L1 therapies have gained approval for the same indication. In these cases, decisions on which drug to use are often left to physician preference, as no data exist on which to base these choices. Many would argue that the efficacy of these therapies is equivocal, at least in terms of overall survival. This is somewhat supported by meta-analyses that attempt to mathematically compare the separate trial data (3). Nevertheless, each of these biologics has a unique binding site on its target molecule and different modifications to the Fc region of the Ab (4–6). Structural modeling has shown that the mechanism by which they bind their targets and the conformation of the molecules once bound differ considerably (5). The degree to which they each block PD-1 signaling varies (7). Furthermore, there are reports of durable responses being achieved by one Ab after a similar agent has failed (i.e., nivolumab after pembrolizumab in melanoma and pembrolizumab after atezolizumab in triple-negative breast cancer) (8, 9). Collectively, this suggests that more careful, direct comparisons of these agents could yield insights that both inform current clinical decisions and lead to better design of the next generation of therapies that target these molecules.

These types of questions are best addressed within the preclinical space, as conducting copious clinical trials to compare the various anti–PD-1/L1 agents is neither feasible nor ethical. Unfortunately, many FDA-approved mAbs targeting PD-1 and PD-L1 do not cross-react between human and mouse proteins. Consequently, preclinical studies are performed using anti-mouse Ab clones that strive to recapitulate the anti-human versions but leave us to wonder what differences, if any, may be at play. For example, it has recently been shown that some anti-mouse PD-1 Ab clones may result in unintended depletion of Ag-specific T cells (10). Thus, it would be advantageous to be able to compare the genuine FDA-approved biologics head to head across various tumor models to determine which would be preferential in certain clinical contexts prior to initiating clinical trials. This would also allow newly developed anti–PD-1/L1 Ab therapeutics or combination strategies to be compared with the currently approved drugs in vivo to further refine clinical trial design.

Thus, we developed “HuPD-H1” mice that express humanized PD-1/L1 sequences to permit the use of anti-human PD-1/PD-L1 Ab therapeutics in vivo. We first show the characterization of this new model at baseline, ensuring that the chimeric proteins function normally and that they successfully bind to anti-human PD-1/L1 therapeutics. Following characterization, we present our comparative studies, with a particular focus on T cell priming in the context of PD-1/L1 inhibition.

It remains a matter of debate as to precisely where and how anti–PD-1/L1 therapies function to produce successful results in responding patients. Recent studies suggest that at least a portion of the antitumor immune response elicited by these therapies occurs through de novo T cell priming in lymphoid organs, perhaps in addition to reinvigorating and expanding exhausted T cell populations at the tumor site (11–13). However, an effect on priming is not feasible to study within human samples. Patients present with malignant disease, and it cannot be determined whether the treatments given are predominantly working at “time zero” of primary Ag exposure versus reactivation of dysfunctional T cells in the periphery.

Hence, with our new model, we sought to compare the strength of the immune response generated by FDA-approved Abs when given during early T cell programming prior to tumor formation. To do this, we combined humanized PD-1/L1 mice with an in vivo priming assay in which anti–PD-1/L1 therapies were administered at the time of T cell priming against a surrogate tumor Ag (OVA), followed by subsequent B16-OVA tumor challenge. Tumor rejection versus formation upon tumor challenge provided a very clear readout of the strength of the immune reaction produced, which differed among the drugs tested. We then defined the specific population of CD8^+^ T cells that correlated with enhanced antitumor immunity, a population that was apparent both in our model and in samples from clinical responders. Taken together, these results demonstrate that HuPD-H1 mice represent a useful tool that will decrease the distance between the preclinical and clinical space and accelerate translational studies to further improve PD-1/L1 therapy.

## Materials and Methods

### Study approval

The Mayo Clinic Institutional Animal Care and Use Committee approved all animal experiments, and mice were maintained under pathogen-free conditions in the animal facility at the Mayo Clinic (Rochester, MN).

### HuPD-1 and HuPD-L1 transgenic mice

The appropriate PD-1 and PD-L1 knock-in targeting vectors containing a neomycin (NEO) selection cassette were electroporated into C57BL/6 FLP embryonic stem (ES) cells. ES cells were then screened for uptake of the targeting vector and NEO cassette deletions. Once positive ES cells were identified, they were microinjected into blastocysts, which were then implanted into pseudopregnant foster mice. The resulting chimeras were mated with wild-type C57BL/6 mice, and F1 heterozygotes were identified from their offspring. Ongoing PCR genotyping of the established mouse lines was completed via EconoTaq Plus Green 2× master mix and the following primer sequences (annealing temperature of 60°C): HuPD-1 forward, 5′-TGGCTCCATACCACAAGCATGG-3′, reverse, 5′-ACTCTGAAGTGTTCCTTGTCCGAGG-3′ (wild-type band expected at 301 bp; when positive for transgene, band expected at 397 bp); HuPD-L1 forward, 5′-AACGTGAGAGCAAGCTTATGCTTCAGG-3′, reverse, 5′-TATCCCTACAATGCCCTGGCCTGG-3′ (wild-type band expected at 277 bp; when positive for transgene, band expected at 367 bp).

Adult mice (2–9 mo of age) were used in all experimental studies. Approximately equal numbers of male and female mice were used.

### Flow cytometry

Cells were stained for flow cytometry assays in FACS buffer (1× PBS, 2 mM EDTA, and 3% FBS). Analysis was completed using a CytoFLEX LX (Beckman Coulter) (DAQ version 2.233, MCB version 3.01) running CytExpert software. The Abs used included anti-mouse CD8-BV421 (clone 53-6.7), anti-mouse CD11a-PerCp/Cy5.5 (clone M17/4), anti-mouse CX3CR1-PE/Cy7 (clone SA011F11), anti-mouse PD-1-allophycocyanin (clone RMPI30), anti-human PD-1-allophycocyanin (clone EH12.2H7), anti-mouse CD11c-BV421 (clone N418), anti-mouse CD4-FITC (RM4-5), anti-mouse CD44-BV711 (IM7), anti-mouse CD45R/B220-BV605 (RA3-6B2), and anti-mouse ICOS-allophycocyanin (C398.4A), all from BioLegend; iTAg MHC tetramer H-2K^b^ OVA and SIINFEKL-PE from MBL International; anti-mouse B220-SupreBright 600 (RA3-6B2), anti-mouse CD4-eFluor 450 (RM4-5), and anti-human PD-L1–PE (clone M1H1), all from Invitrogen; anti-mouse/rat FOXP3-FITC (FJK-16s) from eBioscience; anti-mouse CXCR5-biotin (2G8) from BD Biosciences; and anti-mouse CD8-allophycocyanin/Cy7 (53-6.7) and anti-CD62L–PE-Cy7 (MEL-14) from Tonbo Biosciences. The detailed gating strategies for the assays in Figs. 2 and 5 are shown in Supplemental Fig. 1.

### HuPD-1–activated T cells

Spleens from HuPD-1 homozygous mice or wild-type C57BL/6 mice were processed into single cells and plated in complete RPMI 1640 medium. Cells were activated with anti-mouse CD3/CD28 Dynabeads (Life Technologies, 25 µl of beads per 1 ml of medium). After 48 h of activation, the cells were collected, counted, resuspended in FACS buffer, and stained for flow cytometry.

### HuPD-L1 bone marrow–derived macrophage and spleen cell isolation followed by RT-PCR

Mouse bone marrow–derived macrophages (BMDMs) were prepared as previously described (14). BMDMs were scraped from plates, counted, and replated on day 6 of maturation. A portion of the cells was treated with 10 µg/ml LPS (LPS-EB Ultrapure from InvivoGen). Cells were collected after 24 h of treatment and either 1) stained with anti-human PD-L1–PE (clone M1H1) (Invitrogen) and analyzed via flow cytometry as above or 2) collected in RLT buffer from the Qiagen RNeasy Plus mini kit for later RNA isolation.

Whole spleens from homozygous HuPD-L1 mice and C57BL/6 mice were processed into single cells and plated in complete RPMI 1640 containing 5 µg/ml Con A for activation. These cells were collected at 48 h postactivation in RLT buffer from the Qiagen RNeasy Plus mini kit for later RNA isolation.

Cell samples in RLT buffer were processed via the Qiagen RNeasy Plus mini kit according to the manufacturer’s instructions. Reverse transcription was completed using a SuperScript III reverse transcriptase kit (Invitrogen), followed by PCR with Platinum PCR SuperMix high fidelity (Invitrogen). The following primers were used with an annealing temperature of 55°C and 35 total PCR cycles: mouse PD-L1 forward, 5′-ACCTTAAGCCTCAGCACAGC-3′, reverse, 5′-GTGGCTGGATCCACGGAAAT-3′; human PD-L1 forward, 5′-CCTGGCTGCACTAATTGTCT-3′, reverse, 5′-CAGATGACTTCGGCCTTGGG-3′. cDNA was separated on a 2% gel and visualized under UV light.

### Dendritic cell isolation

HuPD-H1 mice were given a single i.p. injection of 0.5 mg of OVA protein (Sigma-Aldrich) and 50 µg of polyinosinic-polycytidylic acid (poly(I:C); Novus). Untreated HuPD-H1 mice were used as controls. Spleens were harvested 20 h later from both treated and untreated mice, and dendritic cells (DCs) were isolated from total spleen cells using the EasySep mouse pan-DC enrichment kit (STEMCELL Technologies). DCs were then resuspended in FACS buffer and blocked with anti-mouse CD16/32 (clone 93) (BioLegend), followed by staining for flow cytometry with anti-mouse CD11c-BV421 (clone N418) (BioLegend) and anti-human PD-L1-allophycocyanin (clone M1H1) (Invitrogen). One hundred micrograms each of atezolizumab and avelumab was labeled with the Alexa Fluor 647 (AF647) Ab labeling kit (Invitrogen) and used for flow cytometry staining. Flow cytometry analysis was completed as above.

### Receptor occupancy

HuPD-H1 mice were given a single i.p. injection of 0.5 mg of OVA protein (Sigma-Aldrich) and 50 µg of poly(I:C) (Novus). On day 7 postimmunization, pembrolizumab conjugated to AF647 was injected (i.p.) into HuPD-H1 mice. Spleens were collected 24 h later and processed into single cells. A portion of cells was analyzed for AF647 staining without further modification, and a second portion of cells from the same mouse was saturated with labeled pembrolizumab prior to flow analysis (20 µg/ml pembrolizumab-AF647 for 30 min at 4°C, followed by washing with FACS buffer). All cells were stained with anti-mouse CD8-BV421 (clone 53-6.7) (BioLegend), and flow cytometry was completed as above. Receptor occupancy was calculated using the following formula: [(% of total CD8^+^ cells that were pembrolizumab-AF647^+^ after in vivo injection)/(% of total CD8^+^ cells that were pembrolizumab-AF647^+^ after ex vivo saturation)] × 100.

### Cell lines

MC-38 wild-type and MC-38PD-L1KO cells were a gift from Dr. Yang-Xin Fu (15). Human PD-L1 was introduced using a pcDNA3.1 vector with full-length cDNA of human (Hu)PD-L1 (16, 17). MC38HuPD-L1 cells were cultured in complete RPMI 1640 under selection with G4-18 (0.4 mg/ml). B16-OVA cells were a gift from Dr. Richard Vile (Mayo Clinic) and were cultured in complete RPMI 1640 under selection with G4-18 (0.8 mg/ml). B16F10 cells were ordered from the American Type Culture Collection. All cell lines were assessed for mycoplasma contamination via PCR and were negative.

### Tumor experiments

For the MC-38HuPD-L1 study, 0.75 × 10^6^ MC38HuPD-L1 cells suspended in 100 µl of PBS were injected s.c. into the right flanks of HuPD-H1 mice. Mice were checked for tumor growth starting on day 7 after tumor injection and randomized into treatment groups such that each group had a similar average tumor size at start. Perpendicular tumor measurements were taken twice per week by calipers. Drug treatments included 100 µg each of atezolizumab, pembrolizumab, or avelumab injected i.p. in 200 µl of PBS on days 7, 10, 13, 16, and 20 after tumor injection. Two hundred microliters of PBS was injected (i.p.) as a control. Mice were euthanized when tumors grew beyond 200 mm^2^ or when tumors became ulcerated. All remaining mice were euthanized on day 55 after tumor injection. For the tumor growth study comparing PBS versus murine anti–PD-L1 therapy, 0.6 × 10^6^ MC-38 wild-type cells suspended in 100 µl of PBS were injected s.c. into the right flanks of HuPD-H1 mice. Randomization and tumor growth measurements were completed as above. Drug treatment included 100 µg of anti-mouse PD-L1 Ab (clone 10B5) (18) injected i.p. in 200 µl of PBS every 3 d starting at day 6 after tumor injection. Two hundred microliters of PBS was injected (i.p.) into control mice at the corresponding time points.

For all priming experiments, each mouse received a one-time injection (i.p.) of 0.5 mg of OVA protein (Sigma-Aldrich) and 50 µg of poly(I:C) (Novus) in a total volume of 200 µl. Drug treatments included 100 µg each of atezolizumab, pembrolizumab, nivolumab, or avelumab injected i.p. Initial tumor challenge was performed with 1 × 10^6^ B16-OVA tumor cells injected s.c. into the right flank. For rechallenge experiments, mice with no measurable tumors on day 19 after the original B16-OVA injection were rechallenged with 1 × 10^6^ B16-OVA cells in the left flank and 1 × 10^6^ B16F10 cells near the right shoulder. All remaining mice were euthanized at day 14 after rechallenge. To achieve CD8^+^ T cell depletion, 500 µg of anti-CD8 (clone 2.43) (Bio X Cell) was injected (i.p.) on day −1 of the study, followed by 200 µg of anti-CD8 on subsequent injections (days 3, 10, and 17).

### Cytometry by time of flight analysis

HuPDH-1 mice (*n* = 6) received a one-time injection (i.p.) of 0.5 mg of OVA protein (Sigma-Aldrich) and 50 µg of poly(I:C) (Novus) on day 0 of the experiment. In addition, mice received either avelumab (100 µg) or an equivalent volume of PBS injections on days −1, 1, and 3. All mice were given a tumor challenge with 1 × 10^6^ B16-OVA tumor cells injected s.c. into the right flank on day 7. Spleens were then collected on day 10 and processed into single cells in complete RPMI 1640 media (Corning). Cells from each sample were blocked with 1 μg of anti-mouse Fc Block (BD Biosciences) for 10 min at room temperature followed by staining with a mixture of Abs for the T cell panel (Supplemental Table I). Surface marker staining was performed in cell staining buffers (Fluidigm) at room temperature for 30 min. Intracellular cytokine staining was performed using a Cytofix/Cytoperm kit (BD Biosciences) per the manufacturer’s protocol. Upon completion of staining, cells were stored in fresh 1% methanol-free formaldehyde in PBS (Thermo Fisher Scientific) until the day of data collection. All events were acquired on a Helios mass cytometer (Fluidigm). Randomization, bead normalization, and bead removal of the data collected were performed using cytometry by time of flight (CyTOF) software (Fluidigm). Individual FCS (flow cytometry standard) files were exported for analysis and uploaded to the Cytobank software platform (Beckman Coulter) for subsequent analysis. Initial gating via Cytobank used ^191^Ir and ^193^Ir DNA intercalators as well as the event length parameter to discern intact singlets from debris and cell aggregates. Live/dead staining was then used to identify live intact singlets. Then, all CD45^+^ cells were gated, followed by gating for CD3^+^CD8^+^ double-positive cells. This population (CD3^+^CD8^+^) was then used for subsequent viSNE and CITRUS analysis as detailed below.

viSNE/t-SNE (t-distributed stochastic neighbor embedding) is a nonlinear dimensionality reduction algorithm developed based on stochastic neighbor embedding and is available in Cytobank. It uses probability value rather than distance to model the similarity between data points (19). For viSNE analysis, files were categorized into either avelumab-treated (*n* = 3) or PBS-treated (*n* = 3) groups. Twenty-eight markers from the T cell panel were used to build the map, including CX3CR1, CD39, Eomes, TCRβ, Tcf1, CD69, Gata3, RORγT, Tim3, IRF4, CD25, CD28, BATF, PD-1, CD11a, CD62L, CD4, CD73, CD38, CD272, CD161NK1.1, CD11b, Fas, CD223, iNos, CD44, CD11c, and CD45. A total of 173,784 events were extracted by the algorithm using equal sampling of 28,964 events per FCS file. A total of 2000 iterations were completed with a final Kullback–Leibler divergence of 4.79075. Once maps were generated, the relative expression level of each marker of interest could be visualized.

Following viSNE analysis, cell subset abundance and functional marker expression were compared using the CITRUS algorithm in Cytobank (20, 21). As noted by Ben-Shaanan et al. (21), this analysis starts with a pool of single-cell events and iteratively hierarchically clusters them based on the similarity of the expression of subsets of the measured channels. This produces overlapping clusters, with the largest cluster being one encompassing all of the sampled events. The pooled dataset is then split back into its constitutive samples, and the relative abundance of cells in each cluster is computed. We used the self-assembling manifold (SAM) algorithm with a false discovery rate (FDR) of 5% (*p* < 0.05) to determine whether any of the computed clusters of CD3^+^CD8^+^ cells were significantly increased or decreased in abundance when comparing avelumab- versus PBS-treated mice. A total of 20,000 events were sampled per file (total of 120,000 events clustered) with a minimum cluster size of 3%. SAM analysis was run three separate times to ensure reproducibility of the results. Cluster 11994 was significantly increased in the avelumab-treated mice in each iteration.

### Statistical analysis

The statistical tests performed and the exact “*n*” included in each experiment are stated within the figure legends. Statistical analysis included the Gehan–Breslow–Wilcoxon test for survival, the unpaired *t* test to determine differences between two groups, and the log-rank (Mantel–Cox) test to compare time to tumor formation. Statistical analysis was performed using GraphPad Prism v8.4.3 or later, with the exception of Fig. 5E, which was completed using Cytobank software, as above. In graphs, bar height represents the mean, and error bars are the SEM, unless otherwise stated. The number of samples for each group was empirically chosen based on knowledge of intragroup variation and expected effect size. No statistical methods were used to predetermine sample sizes. No data were excluded from the analysis. All experiments were repeatable, and the observations were reproducible. Individual tumor growth curves are shown throughout to allow the reader to assess variability among individual mice in each experiment. To minimize potential confounders in all experiments, mice of different treatment groups were cohoused (mixed among the cages), and the order in which the cages received treatments was varied each week. Laboratory members performing caliper measurements of mouse tumors were blinded to the treatment group.

### Data availability

The datasets generated during the current study are available from the corresponding author upon request.

## Results

### Generation and characterization of humanized PD-1 and PD-L1 mice

**FIGURE 1.**
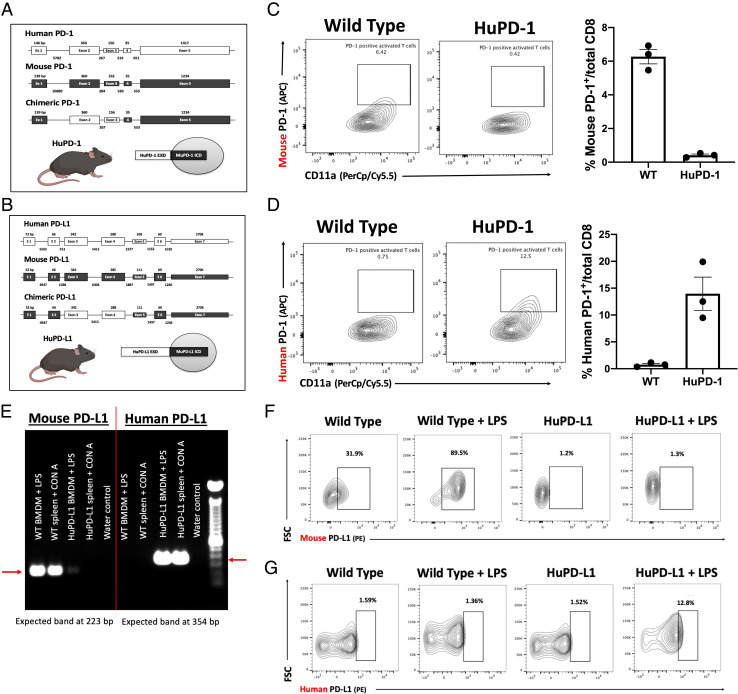
Generation and characterization of humanized PD-1 and PD-L1 mouse strains. (**A** and **B**) Diagrams show the endogenous mouse exons coding for the extracellular domains of PD-1 (exons 2 and 3) or PD-L1 (exons 3 and 4) replaced with the coding sequence for the corresponding human exons to create two separate chimeric strains: HuPD-1 and HuPD-L1. (**C**) Cells from the spleens of adult (3- to 4-mo-old) C57BL/6 wild-type mice or mice homozygous for the human PD-1 knock-in (HuPD-1) were plated and activated with anti-mouse CD3/CD28 beads for 48 h followed by staining with commercial flow cytometry Abs specific for either the mouse or human PD-1 sequence. Cells were first gated for CD8 positivity. Representative flow plots show anti-mouse PD-1 Ab detecting PD-1 on wild-type CD8^+^ T cells, quantified at right (*n* = 3 mice per group; bars indicate mean; error bars indicate SEM). (**D**) Anti-human PD-1 Ab detecting PD-1 on HuPD-1 CD8^+^ T cells, quantified at right (*n* = 3 mice per group). (**E**) RT-PCR primers were designed to detect the mRNA sequences specific to the mouse or human PD-L1 extracellular domain. BMDMs and cells from the spleen of either wild-type or HuPD-L1 mice were treated with LPS or Con A to induce PD-L1 expression. RNA was isolated, and RT-PCR performed. Gel shows bands at expected sizes. (**F**) Representative flow plots show murine PD-L1 expression on the surface of BMDMs from wild-type mice and (**G**) human PD-L1 expression on the surface of BMDMs from HuPD-L1 mice. Increased PD-L1 surface expression is noted following treatment with LPS. FSC, forward scatter.

To generate mice expressing PD-1 and PD-L1 proteins that could be bound by FDA-approved humanized mAbs, we used homologous recombination in ES cells to replace the endogenous mouse exons coding for the extracellular domains of PD-1 (exons 2 and 3) or PD-L1 (exons 3 and 4) with the coding sequence for the corresponding human exons (Fig. 1A, 1B). The remaining mouse exons of each gene were kept intact to ensure normal downstream signals from each molecule within the cytoplasm. These mice were generated on the immunocompetent C57BL/6 background and appeared grossly normal, with no apparent differences in birth rate, growth curves, fertility, or lifespan between the knock-in mice and wild-type C57BL/6 controls (Supplemental Fig. 2A–E).

To evaluate whether our genetic knock-in approach resulted in appropriately expressed and localized proteins, cells from the spleens of C57BL/6 wild-type mice or mice homozygous for the HuPD-1 knock-in (HuPD-1) were plated and activated with anti-mouse CD3/CD28 beads for 48 h to drive PD-1 expression on CD8^+^ T cells. Cells were then stained with commercial flow cytometry Abs specific for either the mouse or human PD-1 sequence. As expected, PD-1 on wild-type mouse CD8^+^ T cells was detected exclusively by the anti-mouse–specific PD-1 clone, whereas PD-1 on HuPD-1 CD8^+^ T cells was detected only by the anti-human–specific PD-1 Ab clone (Fig. 1C, 1D). We did note an increased percentage of CD8^+^ T cells staining positive for PD-1 in the HuPD-1 samples (∼15%) as compared with the wild type samples (∼6%). As it was necessary to use two different clones for staining human versus mouse proteins (clone RMPI30 for mice and clone EH12.2H7 for humans), this is most likely an artifact of varying Ab affinity and binding location. However, it remains a possibility that there is a subtle increase in PD-1 surface expression in the HuPD-1 mice.

Because PD-L1 expression is typically very low in normal tissues at baseline but can be induced on monocytes and macrophages, we next assessed the expression of the chimeric PD-L1 gene in either BMDMs treated with LPS or cells from whole spleens treated with Con A. Using primers specific for the extracellular portions of either mouse or human PD-L1, we detected the mouse version of PD-L1 mRNA in cells from wild-type mice and the humanized version of PD-L1 mRNA in HuPD-L1 mice via RT-PCR (Fig. 1E). Via flow cytometry, mouse PD-L1 protein was detected on the surface of wild-type BMDMs using mouse-specific PD-L1 Ab, whereas human PD-L1 was detected on HuPD-L1 BMDMs using anti-human–specific PD-L1 Ab (Fig. 1F, 1G). In addition, we noted the expected increase in surface expression of murine PD-L1 in wild-type mice following LPS treatment as well as an increase in human PD-L1 in HuPD-L1 BMDMs after the same stimulus, confirming that the chimeric protein is appropriately upregulated by an upstream inflammatory signal (Fig. 1F, 1G). In these studies, there is a notable difference in the level of PD-L1 being detected in the wild-type BMDMs versus BMDMs from the HuPD-L1 mice. As in the PD-1 flow assays, this may be attributed to the necessity of using two different Ab clones with varying affinities to detect mouse versus human protein (clone M1H5 for mice; clone M1H1 for humans). This is plausible given that we found robust expression of humanized PD-L1 mRNA and have no evidence that PD-L1 gene expression has been compromised (Fig. 1E). However, PD-L1 is known to have a binding partner, CMTM6, which stabilizes PD-L1 at the cell surface membrane and affects its internalization and degradation (22). It is possible that the chimeric PD-L1 protein does not bind CMTM6 as efficiently as the murine version and that this results in decreased PD-L1 expression in the humanized mice. This can be explored in future studies. Regardless, the chimeric protein is exclusively expressed in the HuPD-L1 mice, and it is appropriately upregulated following stimulation (LPS). As humanized mice were used as controls in all subsequent experiments, the level of PD-L1 should not differ between control and drug-treated mice at baseline, thus controlling for any possible decreased expression levels that may be present.

**FIGURE 2.**
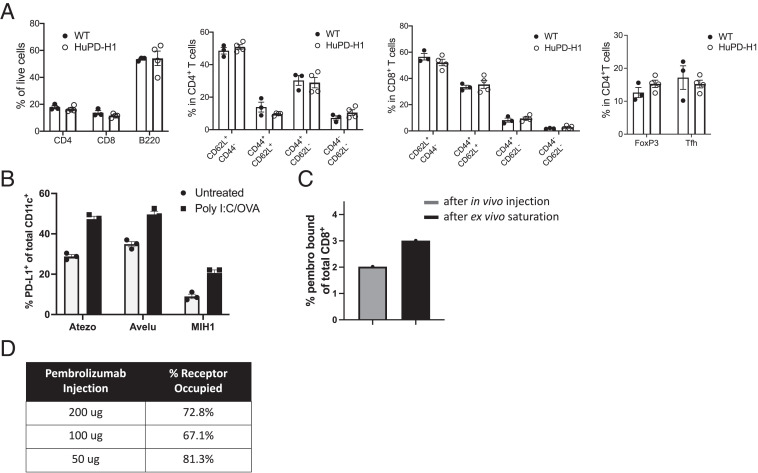
Immune profiling and evaluation of anti-human PD-1 and PD-L1 Ab binding in HuPD-H1 mice. (**A**) Spleens of untreated HuPD-H1 mice and wild-type (WT) C57BL/6 controls were analyzed via flow cytometry for T cell subsets including Treg and T follicular helper and B cell markers. No significant differences were found among the subsets (*n* = 4 HuPD-H1, *n* = 3 WT mice; bars indicate mean; error bars indicate SEM). (**B**) DCs were isolated from the spleens of HuPD-H1 mice 20 h after injection of OVA/poly(I:C) (versus untreated mice). Atezolizumab (Atezo) and avelumab (Avelu) were directly conjugated to AF647 and used to detect surface levels of human PD-L1 via flow cytometry versus commercial anti–PD-L1 clone (M1H1) (*n* = 3 mice/group; bars indicate mean, error bars indicate SEM). (**C**) In vivo receptor occupancy of PD-1 by pembrolizumab. Gray bar is percent of total CD8^+^ T cells bound by pembrolizumab-AF647 24 h after in vivo injection. Black bar represents percent of total CD8^+^ T cells from same mouse when saturated with pembrolizumab-AF647 ex vivo. The percent difference between these two values is the receptor occupancy. Bar graph is from 100-µg in vivo injection. Results from three different injection concentrations shown in (**D**) (*n* = 3 mice).

Next, we produced double knock-in mice that were homozygous for both HuPD-1 and HuPD-L1 sequences. These mice were used in all subsequent experiments and are referred to as HuPD-H1. We compared HuPD-H1 mice with wild-type C57BL/6 controls to ensure that there were no differences in the relative proportions of lymphocyte subsets at baseline. The relative proportions of T cells (CD4^+^, CD8^+^) and B cells (B220^+^) were normal in spleens from HuPD-H1 mice, and the relative proportions of naive (CD62L^+^CD44^−^), central memory (CD62L^+^CD44^+^), and effector (CD62L^−^CD44^+^) T cell populations were also equivalent to those in wild-type mouse spleens. Finally, we evaluated baseline levels of regulatory T (Treg) (CD4^+^B220^+^Foxp3^+^) and T follicular helper (CD4^+^CXCR5^+^ICOS^+^) cells in the spleen at baseline and found that these populations were also unchanged in HuPD-H1 mice when compared with wild-type controls (Fig. 2A).

Then, we sought to ensure that FDA-approved anti-human PD-L1 therapies could bind to chimeric PD-L1 protein expressed by HuPD-H1 mice. To test this hypothesis, we injected HuPD-H1 mice with OVA/poly(I:C) to increase PD-L1 expression in DCs (versus untreated littermates) and then isolated DCs from the spleens 20 h later. Atezolizumab and avelumab were directly conjugated to AF647 so that they could be detected via flow cytometry. The commercial flow cytometry Ab clone M1H1, which is typically used to detect PD-L1 on human cells, was used as a positive control. The results showed that atezolizumab and avelumab were both able to bind PD-L1 on HuPD-H1 DCs (Fig. 2B). As expected, there was an increased level of PD-L1 staining on DCs isolated from mice treated with poly(I:C)/OVA compared with untreated mice, confirming the specificity of Ab–PD-L1 binding (Fig. 2B).

We next considered whether FDA-approved anti–PD-1 therapy could bind to PD-1 in the HuPD-H1 model and assessed this in vivo via a pharmacologic assay known as “receptor occupancy.” Mice were first given an injection of OVA protein combined with poly(I:C) to stimulate an immune reaction. On day 7 postimmunization, pembrolizumab conjugated to AF647 was injected i.p. into HuPD-H1 mice at varying concentrations. Spleens were collected 24 h later, and the percentage of CD8^+^ T cells bound by pembrolizumab was quantified by flow cytometry. This was further compared with a portion of cells from the same spleens that were saturated with labeled pembrolizumab ex vivo, allowing us to quantify the maximum amount of PD-1 that could be bound versus what had been bound in vivo. In our model, PD-1 receptor occupancy on CD8^+^ T cells ranged from 67.1 to 81.3% and was not dose-dependent, in agreement with the pharmacokinetic data of pembrolizumab and nivolumab in the limited human studies that have assessed this metric (23, 24) (Fig. 2C, 2D).

**FIGURE 3.**
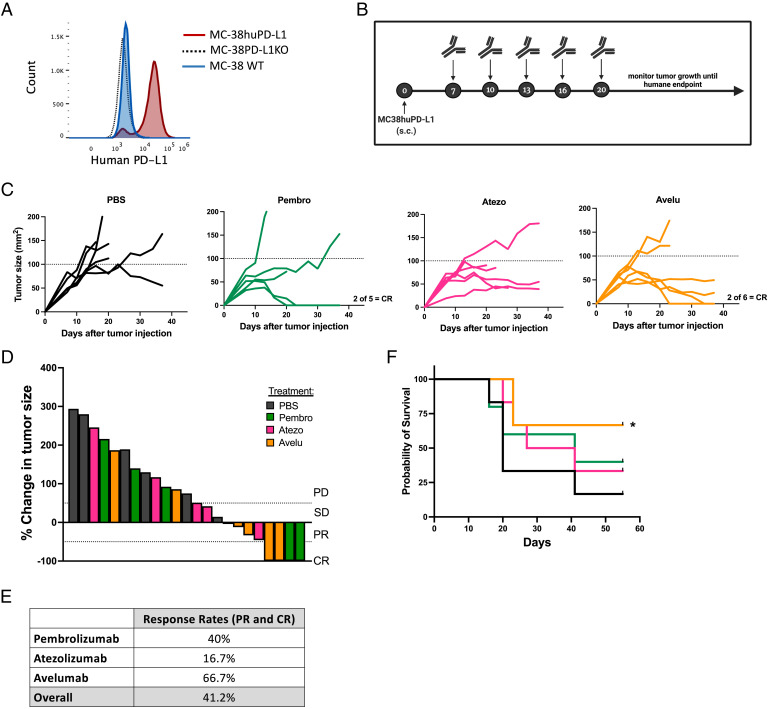
Therapeutic effects of FDA-approved anti–PD-1/L1 Abs in HuPD-1-H1 mice. (**A**) Human PD-L1 expression confirmed on MC-38HuPD-L1 cells (M1H1 clone used for detection). (**B**) Schematic of experimental timeline. Ab was injected (i.p.) at 100 μg per mouse at each time point. (**C**) Individual tumor growth curves (*n* = 6 PBS, *n* = 5 pembrolizumab [Pembro], *n* = 6 atezolizumab [Atezo], *n* = 6 avelumab [Avelu]). CR, complete responder. (**D**) Calculated percent change in tumor size from onset of treatment at day 7 until time of death. CR, complete response (more than −100% decrease); PD, progressive disease (>50% increase in tumor size); PR, partial response (−1 to −50% decrease); SD, stable disease (0–50% increase). Each bar represents an individual animal. (**E**) Calculated response rates based on data in (D). (**F**) Survival curve (*p < 0.05 avelumab versus PBS, Gehan–Breslow–Wilcoxon test).

As MC-38 mouse colon carcinoma tumors are known to be sensitive to PD-1/L1 blockade therapy in preclinical models that use anti-mouse PD-1/L1 Ab clones (15, 25), we next confirmed that MC-38 tumors in HuPD-H1 mice remained sensitive to anti-mouse PD-L1 Ab (clone 10B5) (Supplemental Fig. 2F, 2G). We then created MC-38HuPD-L1 cells in which the mouse PD-L1 sequence was removed and replaced by the human PD-L1 sequence, thus making both the host and tumor cells a fully humanized PD-1/L1 system ideal for testing the FDA-approved therapies (Fig. 3A). Following s.c. injection of MC-38HuPD-L1, mice began receiving anti-human PD-1/L1 Abs (i.p.) on day 7 of tumor growth (Fig. 3B). Tumor responses were variable across individual mice (Fig. 3C). Although a number of tumors continued to grow despite treatment, a subset responded, with three mice achieving a partial response and four mice experiencing a complete response (two in the pembrolizumab group and two in the avelumab group) (Fig. 3D). In total, 7 of 17 mice achieved either a partial response or complete response across all three drug treatments, reflecting an overall response rate of 41.2% (Fig. 3E). The highest response rate was achieved by avelumab (66.7%), which resulted in a statistically significant increase in overall survival in the avelumab group (Fig. 3F). It is notable that our fully humanized model has a less consistent response rate than many of the fully murine-based MC-38 models and anti-mouse Ab therapeutics typically used for preclinical studies. However, this is not without precedent. Single knock-in, humanized PD-1 mice had a mixed response to pembrolizumab in a previous study (26). Furthermore, these more variable responses are more in line with the ∼30% response rate seen in human clinical trials over time and may reflect the more complex immunological mechanisms at play in human disease. Thus, in this regard, the humanized PD-1 and PD-L1 mouse model recapitulates the therapeutic effects of FDA-approved PD-1 or PD-L1 Abs in vivo.

### Direct comparison of the antitumor immune response generated by FDA-approved anti–PD-1/L1 Abs in an in vivo T cell priming model

**FIGURE 4.**
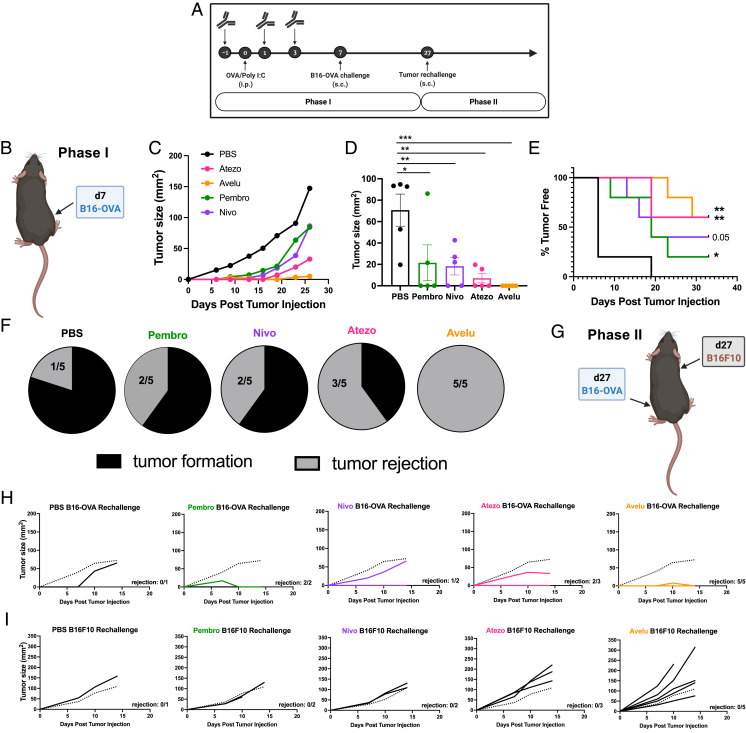
FDA-approved Abs promote tumor Ag-specific vaccination therapy. (**A**) Schematic of two-phase experimental timeline. (**B**) Initial B16-OVA tumor injections were in right flank. (**C**) Averaged tumor growth curves of day 7 B16-OVA tumors (*n* = 5 mice per group, dot represents mean). (**D**) Tumor size comparison at day 19 of tumor growth (unpaired *t* test). Bars indicate mean; error bars indicate SEM (*p < 0.05, **p < 0.01, ***p < 0.001). (**E**) Time to tumor formation (each group compared with PBS, log-rank [Mantel–Cox] test). (**F**) Fraction of experimental mice (in C–E) that completely rejected day 7 primary B16-OVA tumor challenge. (**G**) Mice without primary B16-OVA tumor growth were rechallenged at day 27 with B16-OVA on the opposite (left) flank as well as B16F10 over the right shoulder. (**H** and **I**) Individual tumor growth curves for all rechallenged mice. Dotted line shows average growth of B16-OVA or B16F10 cells in completely naive mice. All remaining mice were euthanized at day 14 after tumor rechallenge.

Recent evidence suggests that the antitumor effects of PD-1/L1 Ab therapies are transpiring in the periphery in connection with T cell priming (11–13). This has implications for how to optimize typical, single-agent treatment regimens, but also indicates that combining anti–PD-1/L1 therapeutics with tumor vaccines should be efficacious. A recent trial by Ott et al. (27) treated patients for 12 wk with anti–PD-1 therapy prior to administering the NEO-PV-01 vaccine, which resulted in durable neoantigen-specific T cell reactivity. Preclinical studies suggest that vaccination could “deepen” the immune response for those already showing a response to immunotherapy alone (28). Thus, we sought to directly compare the strength of the immune response generated if each FDA-approved Ab therapeutic was present at the time of T cell priming within our in vivo model. To do this, we i.p. administered anti–PD-1/L1 Ab therapeutics during an initial T cell priming window against a surrogate tumor Ag (OVA). The treatment was then discontinued, and the strength and durability of the systemic T cell response were evaluated with a s.c. tumor challenge distant from the T cell priming site (Fig. 4A).

Mice were treated with PBS, pembrolizumab, nivolumab, atezolizumab, or avelumab on days −1, 1, and 3, surrounding an immunization with OVA protein and poly(I:C) adjuvant on day 0 (Fig. 4A). One week after immunization, mice were challenged with a s.c. injection of B16-OVA mouse melanoma tumor cells in the right flank (Fig. 4B). Most mice in the PBS-treated group had visible tumors by day 6 after tumor injection (Fig. 4C–E*;* black lines). In contrast, mice that were primed with OVA in the presence of anti–PD-1/L1 therapies displayed a much greater capacity to delay tumor formation or prevent it altogether. Interestingly, the two anti–PD-L1 therapies had the strongest effect, with three out of five atezolizumab-treated mice and five out of five avelumab-treated mice rejecting tumor formation out to day 19 after tumor injection (Fig. 4F).

To test the durability of this protective antitumor response, we rechallenged all of the mice that had no signs of primary tumor growth 3 wk after tumor injection with a second injection of B16-OVA tumor cells at a distant site (this time on the left flank, (Fig. 4G). This rechallenge occurred a total of 4 wk after the last drug treatment. Most of the mice that were OVA immunized in the presence of anti–PD-1/L1 blockade were able to reject a second tumor challenge at a distant site, including all five of the mice initially primed with avelumab (Fig. 4H). To determine whether this was an Ag-specific effect, we also challenged the mice with an injection of B16F10 (non-OVA expressing) tumor at a third location. The B16F10 tumors grew out in all mice (Fig. 4I), indicating that the rejection of B16-OVA was due to an Ag-specific adaptive immune response. Collectively, these results show that FDA-approved anti–PD-1/L1 mAb therapeutics modulate T cell priming to generate durable immunity against tumor formation and that these effects can be directly compared in the HuPD-H1 model.

### Anti–PD-L1 Ab increases the expansion of Ag-primed, cytotoxic CD8^+^ T cells for protective immunity

In our in vivo model, all mice were primed with OVA and poly(I:C) to generate an Ag-specific CD8^+^ T cell response. We confirmed that OVA-specific CD8^+^ T cells were produced equivalently in both PBS- and anti–PD-1/L1-treated mice (Supplemental Fig. 3A, 3B). In both groups, this response peaked at day 7 after immunization, with decreasing amounts of OVA tetramer^+^CD8^+^ T cells over time. However, we found no significant difference in the abundance of OVA-specific tetramer^+^CD8^+^ cells between controls and drug-treated mice at any of the time points we assessed. Hence, despite the clear presence of Ag-specific cells within the PBS mice, these cells failed to prevent eventual tumor growth. This was in contrast to the mice primed in the presence of anti–PD-1/L1 (Fig. 4C–E).

**FIGURE 5.**
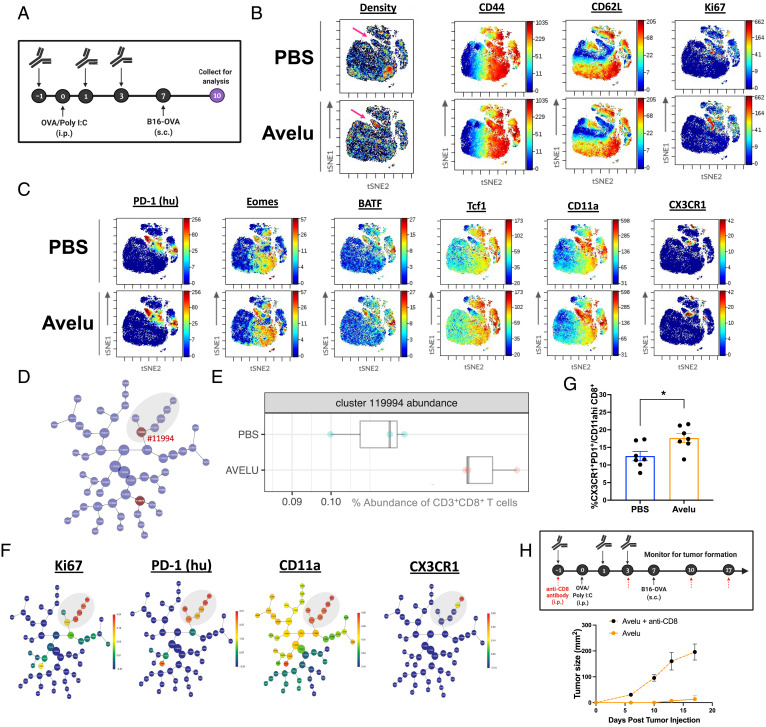
Anti–PD-L1 Ab increases the expansion of Ag-primed cytotoxic CD8^+^ T cells for protective immunity. (**A**) Schematic of experimental timeline. (**B** and **C**) Cells from spleen were isolated and T cell marker expression was captured via CyTOF. High dimensional data were analyzed via Cytobank software’s viSNE algorithm. CD3^+^CD8^+^ T cells from representative PBS and avelumab samples are shown (*n* = 3 PBS, *n* = 3 avelumab analyzed total). Density gradient indicates a population more prominent in the avelumab-treated mice (pink arrows). Additional plots display relative expression of various T cell markers. (**D**) In a separate analysis, CITRUS using SAM with a 5% FDR was completed using all CD3^+^CD8^+^ T cells to detect differences in the abundance of T cell subsets in PBS versus avelumab-treated mice. Parent cluster 119994 (highlighted in red, with progeny clusters shaded in gray) had a significantly higher abundance in avelumab-treated mice. (**E**) Cluster abundance shown as boxplot (*n* = 6 separate CITRUS with SAM repeats produced similar results). (**F**) CITRUS tree with expression of markers overlaid indicates high expression (red) to low expression (blue) of each marker. Gray shading shows parent cluster 119994 and progeny, as in (D). (**G**) Flow cytometry completed on separate samples confirms CyTOF findings (*n* = 7 mice/group, experimental timeline as in A). Bars indicate mean; error bars indicate SEM (*p < 0.05). (**H**) Above, Schematic of experimental timeline. Below, Average tumor growth curves for each group (*n* = 6 in avelumab group; *n* = 5 avelumab + anti-CD8 treatment group; dots indicate mean; error bars indicate SEM). Avelu, avelumab.

To explain this, we next considered whether treatment with FDA-approved therapies at the time of Ag stimulus led to a shift in the phenotype of the resulting T cells such that they were more functionally able to control tumor seeding and growth and whether this could account for the more effective and durable antitumor immunity within those mice. For these experiments, we focused on PBS control versus avelumab-treated mice, as avelumab had the most robust effect in the previous in vivo challenge experiments. We isolated spleens from mice primed with avelumab (versus PBS) and compared the phenotype and abundance of CD8^+^ T cells 72 h after B16-OVA tumor injection (day 10 overall) with a large panel of T cell markers via CyTOF (Fig. 5A, Supplemental Table I). We then performed viSNE dimensionality reduction analysis using equal numbers of CD3^+^CD8^+^ T cells as input and produced t-SNE maps that distributed the cells based on their phenotype (level of marker expression). We noted an increased density of cells near the center of the map that was specific to the avelumab-treated mice (Fig. 5B, pink arrows). The overlay of T cell marker expression onto the maps revealed that this subpopulation was CD44^hi^CD62L^lo^ (effector T cells). They were also Ki67 positive, indicating proliferation at the time of tumor challenge. Furthermore, the cells were relatively high in PD-1 expression, with moderate expression of the transcription factor Eomes, indicating that they were differentiated and Ag primed (29, 30) (Fig. 5C). They moderately expressed BATF, a transcription factor shown to counter T cell exhaustion (31). Conversely, the population of interest was relatively low in Tcf1 expression. Previously, PD-1^+^Tcf1^lo^ cells have been characterized as fully differentiated, effector T cells that are produced from Tcf1^hi^ cells and promote tumor control in response to ICB therapy (32). Finally, we noted that the subset of interest in the avelumab-treated mice displayed noticeably high expression of CD11a and CX3CR1 (Fig. 5C).

Interestingly, the CD11a^hi^CD8^+^ T cell population has been used previously as a surrogate for total Ag-primed T cells in both mice and humans (33). In addition, our group was the first to discover increased expression of the chemokine receptor CX3CR1 in the CD8^+^ T cells of successful responders to anti–PD-1 treatment (34). Since that time, the CX3CR1^+^ CD8 T cell population has been found to increase in responders after treatment in a number of studies, including models of both tumor and chronic viral infection (35–38). Taken together, our results from the HuPD-H1 model show that expansion of the CX3CR1^+^CD8^+^ T cell subset can be induced by the presence of ICB therapy at the time of T cell priming, and an increase in these cells is correlated with enhanced tumor rejection in the anti–PD-1/L1-treated mice.

To further verify these results, we completed an additional analysis of the CyTOF data using CITRUS, an algorithm that iteratively hierarchically clusters cells based on similarity of expression of subsets of the measured channels. This produced a map of nodes (a tree) with parent nodes upstream of their relative progeny (Fig. 5D). Each node contains cells with similar phenotypes based on marker expression. Statistical analysis (FDR of 5%) found that cluster (or node) 119994 was significantly more abundant in the avelumab-primed mice than in the controls (Fig. 5E). Overlay of the relative expression of various markers onto the CITRUS map revealed that cluster 119994 had high expression of Ki67, PD-1, CD11a, and CX3CR1 (Fig. 5F). This, therefore, represents the same population of therapy-responsive cells initially found via the t-SNE maps.

To validate our CyTOF findings in an expanded number of samples, we used flow cytometry to show that mice primed in the presence of avelumab had significantly more CD11a^hi^CX3CR1^+^PD-1^+^CD8^+^ cytotoxic T cells at day 10 post-OVA immunization than control mice (Fig. 5G). In addition, there was a trend toward an increased frequency of CX3CR1^+^PD-1^+^ cells among the tetramer^+^ (SIINFEKL-specific) CD8^+^ T cells of the avelumab-treated mice (Supplemental Fig. 3C, 3D). However, this did not reach statistical significance. Given that the CD11a^hi^ population contains tetramer^+^ cells as well as additional Ag-primed T cells to other OVA Ags beyond just SINFEKL (Supplemental Fig. 3E) (33), this result may imply that T cells primed against other, less dominant OVA Ags are important in the overall shift toward a more cytotoxic T cell response.

Finally, to confirm that tumor rejection induced by OVA/poly(I:C) plus avelumab was indeed a CD8^+^ T cell–dependent effect, we depleted CD8^+^ T cells starting on day −1 at the time of the first drug treatment. We found that mice primed against OVA, but without CD8^+^ T cells, were unable to reject the B16-OVA tumor challenge, even if priming took place in the presence of avelumab (Fig. 5H). Collectively, our results indicate that ICB therapy at the time of T cell priming can induce expansion of a unique effector-like CD8^+^ T cell population capable of tumor rejection. This population correlates with tumor rejection both in our model and in patient clinical samples (34, 38).

## Discussion

Although the current FDA-approved anti–PD-1 or PD-L1 blocking Abs have demonstrated encouraging clinical responses, their underlying mechanisms of action still need to be better defined, particularly for optimizing therapeutic timing and identifying new opportunities for rational combination therapy. To that end, we successfully generated humanized PD-1 and PD-L1 mice in which the human sequences of the extracellular domains of PD-1 and PD-L1 were used to replace both mouse PD-1 and PD-L1. Unlike current preclinical models that use anti-mouse Ab surrogates or immunocompromised mice with human-derived bone marrow, our HuPD-H1 mice allow direct comparison of FDA-approved anti–PD-1 and anti–PD-L1 therapeutics within fully immunocompetent C57BL/6 mice.

Although the ultimate safety and efficacy of immune checkpoint inhibitors (ICIs) are best defined in human clinical studies, preclinical models are still needed to understand the biological impacts of these therapies that cannot be easily identified from patient samples. For example, the use of CTLA-4 humanized mice (39) was essential for delineating the true mechanism of anti–CTLA-4 Abs in vivo. Using the FDA-approved anti–CTLA-4 Ab that binds to human but not mouse protein, researchers were able to determine that binding of CTLA-4 efficiently induces Treg cell depletion and Fc receptor-dependent tumor rejection, rather than the previously accepted mechanism of blocking the interaction of B7 and CTLA-4. This observation not only explains why anti–CTLA-4 Abs can cause severe colitis, which is usually controlled by Treg cells, but also provides a new platform for evaluating the next generation of therapeutic tools targeting CTLA-4 in vivo. Our humanized PD-1/L1 mouse model was created to serve a similar purpose in characterizing both current and future anti–PD-1/L1 therapeutics, including not only Abs but also the novel small molecules being developed to target human PD-1/L1 signaling (40).

To date, there has been one previous study using a single knock-in humanized PD-1 mouse model (26). This allowed comparison of anti-human PD-1 therapeutics in immunocompetent mice and yielded novel insights. Building on this, we considered evidence that the PD-1/PD-L1 pathway exhibits bidirectional signaling in the context of tumor–T cell interactions or other immune cell–T cell interactions (41–44). Therefore, to best address the role of anti-human PD-1 or PD-L1 Abs in vivo, we generated a model that incorporated both humanized PD-1 and PD-L1, ensuring the appropriate physiological ligation between the two molecules. Thus, by combining humanized PD-1 and PD-L1 in our HuPD-H1 mice, we now have a unique tool to address numerous questions surrounding these molecules and the therapies that target them. In the current study, we focused on further delineating the biological impact of anti-human PD-1 or PD-L1 Abs during T cell priming, a biological process that encompasses multiple levels of immune cell interactions engaging the PD-1 and PD-L1 signaling pathways (45–47). Our results demonstrate that FDA-approved anti–PD-1/L1 Abs affect early T cell programming, resulting in a sustained effector T cell response capable of eliminating a subsequent tumor challenge. This highlights the potential of combining tumor vaccine therapy and anti–PD-1/PD-L1 Abs to prevent tumor recurrence, which should be further explored in clinical trials.

Another advantage of our double humanized PD-1 and PD-L1 mice was its faithful recapitulation of the clinical relevance of CX3CR1^+^CD8^+^ T cells in response to anti–PD-1 or PD-L1 therapy. We and others have previously reported that CX3CR1^+^CD8^+^ T cells increase in the peripheral blood of responders to ICI therapy for patients with advanced melanoma or lung cancers (34, 38). However, it is still not clear how these PD-1 therapy-responsive CX3CR1^+^CD8^+^ T cells are generated and expanded in patients. In this study, we found that ICIs may expand CX3CR1^+^CD8^+^ T cells shortly after T cell priming and that this subset of T cells shares many features of the effector memory phenotype (BATF^+^ and Eomes^+^TCF-1^−^). Our previous studies also showed that effector CD8^+^ T cells need CX3CR1 for their accumulation at tumor sites (34). Thus, the increase of CX3CR1^+^CD8^+^ T cells within the spleen during T cell priming in the presence of ICI therapy may correlate with an increased migratory capacity of effector CD8^+^ T cells eventually facilitating their enrichment at tumor sites to cause tumor regression. This can be explored in future experiments However, the current study would suggest that the quality of Ag-specific T cells rather than the quantity of Ag-specific T cells plays a critical role in response to ICI therapy, as all mice in our study, including controls, had a robust tumor-specific T cell immune response to the OVA Ag, but only in mice treated with an ICI at the time of priming showed effective antitumor immunity.

In this study, we report initial studies characterizing the HuPD-H1 model, but it is our hope that this new tool will be used by many to address questions not previously possible to explore. For example, in 2016 pembrolizumab received FDA approval as a first-line treatment for stage III or metastatic non–small cell lung cancer, whereas nivolumab did not meet its primary endpoint. Some have attributed the difference in trial outcomes data to criteria limiting the patients in the pembrolizumab study to only those with high tumor expression of PD-L1 (>50%) versus the nivolumab trial, which accepted a less stringent population (>5% PD-L1 expression) (48, 49). However, different companion diagnostics were used to measure PD-L1 expression in the two trials, making the data very difficult to compare. Thus, one could imagine using the Hu-PDH1 mice and lung tumor models with varying degrees of PD-L1 expression to truly delve into the differences in effect between these two anti–PD-1 drugs. In addition, anti–PD-1/L1 Abs with novel mechanisms of action have been reported in the preclinical literature. Rather than blocking PD-1/L1 interactions, these Abs may bind other portions of the proteins and indirectly result in decreased SHP-2 phosphatase recruitment to CD28 or lead to PD-L1 downregulation (17, 26). It will be fascinating to compare these and other new mAb candidates to the currently approved drugs to determine advantages and disadvantages across tumor types and treatment strategies prior to initiating phase I trials.

Although the HuPD-H1 model is a promising new tool for use in advancing anti–PD-1/L1 science and therapeutic applications, biological differences between mice and humans will always be a limitation of preclinical studies. This is true, particularly with the accelerated timeline for tumor growth and treatment, which happens over only a few weeks in mice but over months and years in the clinical setting. In addition, we used the model Ag OVA in our studies along with a strong TLR-3 adjuvant, poly(I:C). The effects of anti–PD-1/L1 therapies in combination with different strengths of Ag and various adjuvants will need to be tested across different tumor types to determine in what setting this combination is most effective for potential vaccine therapy. Because human IgG therapeutics are being administered to immunocompetent mice in this model, anti-human IgG Abs are a concern that could limit therapeutic efficacy in longer-term models. However, studies using human i.v. Ig in mice would suggest this may not be relevant until at least 9 wk of treatment (50). Finally, although we found increased levels of CX3CR1^+^PD-1^+^CD8^+^ effector T cell levels after priming in the presence of avelumab, we have not determined whether these cells are derived directly from the APC/T cell interaction or from an event farther downstream in T cell proliferation and expansion. The existence of “effector precursor” CD8^+^ T cells, initially primed in lymphoid tissues, that then proliferate and produce CX3CR1^+^CD8^+^ T cells upon treatment with PD-1 blockade is a possibility (36). It will be important to use this new model to further parse the origin of CX3CR1^+^ cells, given their tight correlation with successful treatment response in humans.

In summary, our double humanized PD-1 and PD-L1 mice created on the immunocompetent C57BL/6 background provide an in vivo model to address the mechanisms of action of drugs aimed at human PD-1 or PD-L1. In the future, these mice will serve as a platform to evaluate rational combination treatments with chemotherapy, targeted small molecules, or radiation therapy, with the advantage that many syngeneic mouse tumor models have already been established on the C57BL/6 strain. In addition, the recapitulation of PD-1 therapy-responsive T cells in our model also suggests that future studies using these mice could reveal other immune cell biomarkers associated with and critical for the response to PD-1/PD-L1 inhibitors.

## Supplementary Material

Supplemental Figure 1 (PDF)
