# Supplementary material for: A Novel Humanized PD-1/PD-L1 Mouse Model Permits Direct Comparison of Antitumor Immunity Generated by Food and Drug Administration–Approved PD-1 and PD-L1 Inhibitors
Source: Immunohorizons. 2023 Jan 19;7(1):125–39. doi: 10.4049/immunohorizons.2200054 (PMC10106088; doi:10.4049/immunohorizons.2200054)
Supplement: Supplemental Figure 1 (PDF) [file IH_2200054_Supplemental_1.pdf]

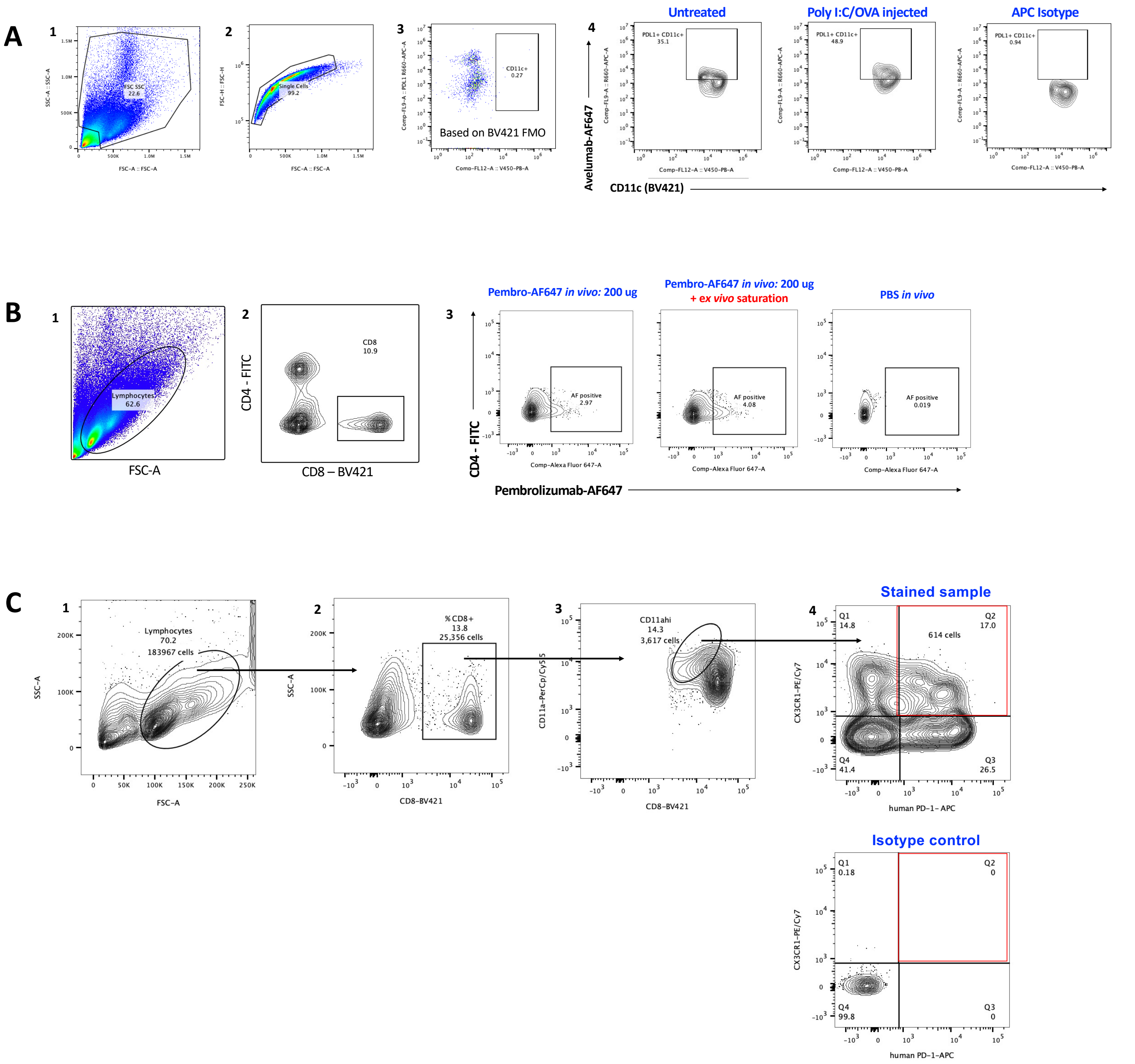

Supplemental Figure 1. *Representative flow cytometry gating strategies for results shown in Figure 2 and Figure 5.*

(**A**) Gating strategy used for analysis of the samples shown in Figure 2B. Total cells were gated for single cells based on morphology and scatter properties, and then CD11c^{+} cells were selected. From the CD11c positive cells, final gating for the Avelumab-AF647 positive cells was completed. Isotype control was used to set positive/negative gate for AF647. (**B**) Gating strategy for analysis of the samples in Figure 2C. Total cells were divided into CD4^{+} vs. CD8^{+} populations. All CD8^{+} cells were then evaluated for pembrolizumab-AF647 staining. A sample from a mouse injected with PBS was used to set positive/negative gate for AF647. (**C**) Gating strategy for analysis shown in Figure 5G with CX3CR1^{+}PD-1^{+}CD11ahiCD8^{+} T cells quantified in final gate. Isotype control is shown below to indicate how positive vs. negative gates were derived.

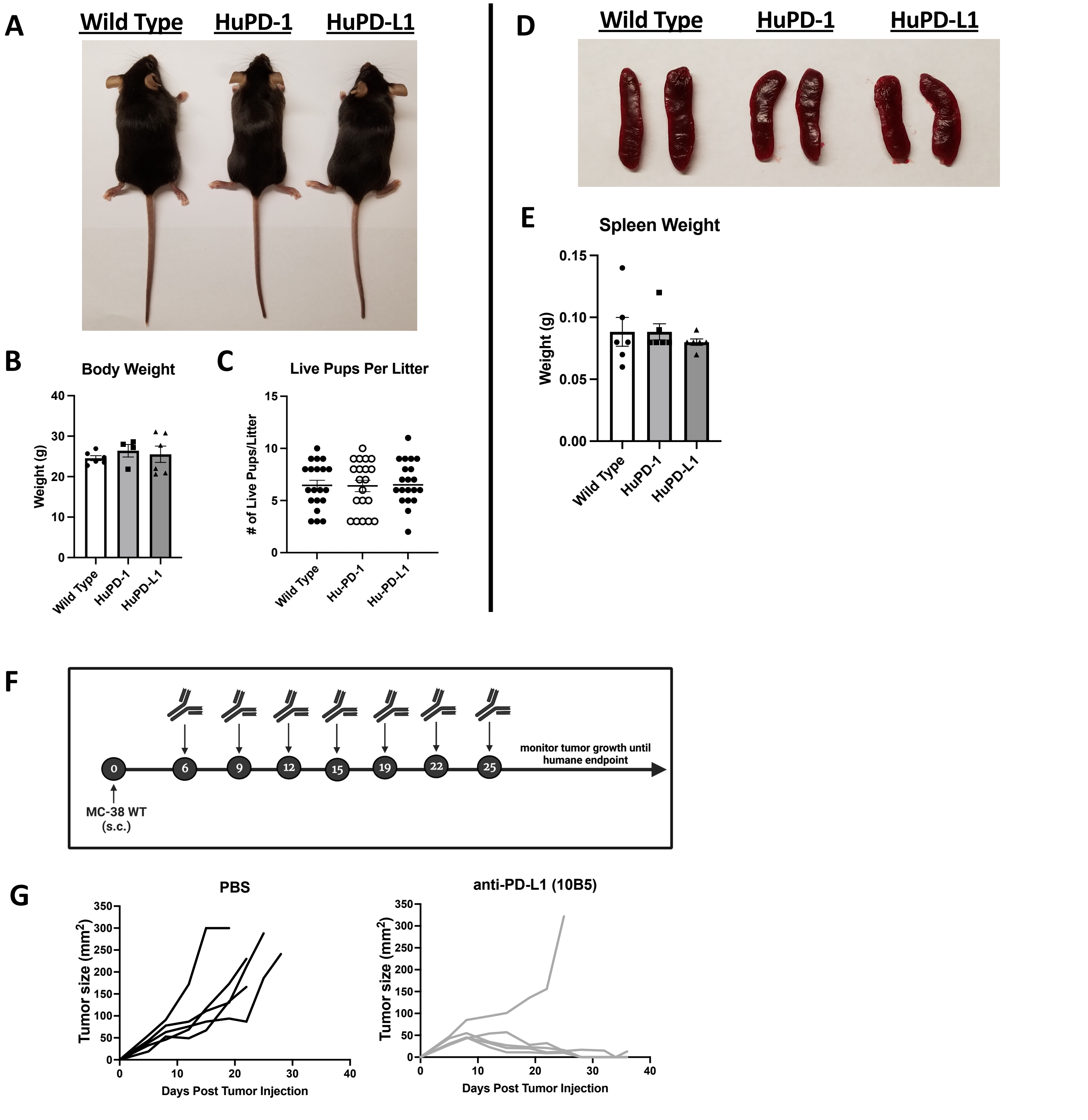

Supplemental Figure 2. *Further characterization of humanized knock-in mice revealed no gross differences when compared to wild type C57BL/6 controls, and MC-38 wild type tumors expressing murine PD-L1 remain sensitive to anti-mouse PD-L1 antibody (clone 10B5) when grown in HuPD-H1 mice.*

(**A-E**) Comparison of 4 to 9-month-old adult mice homozygous for each knock-in gene shown. (**A**) HuPD-1 and HuPD-L1 were normal size, absent of any dermatologic or anatomic abnormalities, (**B**) maintained normal body weight, (**C**) and had no significant difference in fertility or production of live offspring when compared to C57BL/6 mice bred within the same facility. (**D, E**) Gross spleen appearance and spleen weight were also equivalent. Gross spleen samples collected from two mice of each group are shown; spleen weights shown are n=8 from each group. (**F**) Schematic of experimental timeline in HuPD-H1 mice. Antibody was injected (i.p.) at 100 ug per mouse at each indicated time point. (**G**) Individual tumor growth curves (n=5 PBS, n=5 anti-PD-L1 (clone 10B5)).

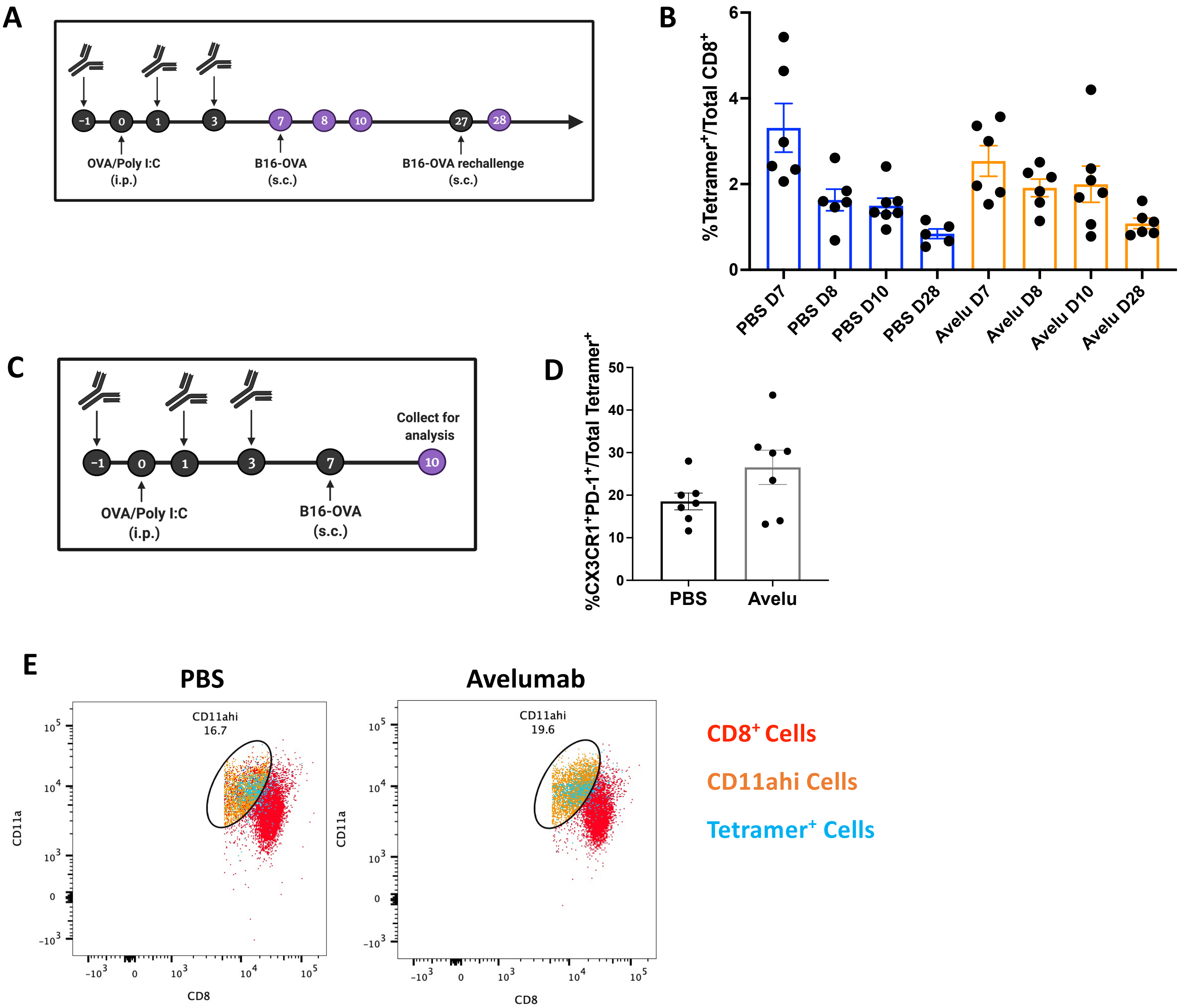

Supplemental Figure 3. *There is no significant increase in Tetramer$^+$CD8$^+$ T cells in PBS vs. avelumab treated mice; further analysis of CX3CR1$^+$PD-1$^+$ population among Tetramer$^+$ cells.*

(**A**) Schematic of experimental timeline. Spleens were collected for analysis at days 7, 8, 10, and 28. (**B**) Percent Tetramer$^+$ cells from total CD8$^+$ T cells quantified by flow cytometry at indicated timepoints (n=6 PBS, n=6 Avelu for each timepoint). No significant difference between groups (bars, mean; error bars, s.e.m.). (**C**) Schematic of experimental timeline, as in Figure 5 of the main text. (**D**) Samples shown in main text Figure 5G were re-analyzed, gating first for all CD8$^+$Tetramer$^+$ cells, and then determining what percent of the Tetramer$^+$ cells were CX3CR1$^+$PD-1$^+$. Results are shown. The increase percentage of CX3CR1$^+$PD-1$^+$ cells in avelumab-treated mice did not reach statistical significance ($p = 0.0992$, student t test; n = 7 mice/group; bars, mean; error bars, s.e.m). (**E**) Notably, the majority of Tetramer$^+$ cells are contained within the CD11ahi gate. Representative flow plots show here contain all CD8$^+$ T cells (red), overlaid with the CD11ahi population (orange) and the Tetramer$^+$ population (blue). Thus, analysis of CD11ahi population, as in Figure 5G is likely reflective of both Tetramer$^+$ cells as well as antigen-primed CD8$^+$ T cells that are reactive to additional OVA-antigens, other than SIINFEKL.

No.	Sp.	Label	Target	Location	Antibody per test (µL)
1	Ms	089Y	CD45	Surface	0.50
2	Ms	110Cd	CX3CR1	Surface	0.25
3	Ms	141Pr	CD39	Surface	1.00
4	Ms	143Nd	TCRb	Surface	0.25
5	Ms	145Nd	CD69	Surface	0.50
6	Ms	149Sm	CD366 (Tim-3)	Surface	1.00
7	Ms	151Eu	CD25 (IL-2R)	Surface	1.00
8	Ms	152Sm	CD3e	Surface	0.25
9	Ms	153Eu	CD28	Surface	1.00
**10**	**Hu**	155Gd	PD-1	Surface	1.00
12	Ms	158Gd	CD11a	Surface	1.00
13	Ms	160Gd	CD62L	Surface	1.00
14	Ms	163Dy	CD4	Surface	0.50
15	Ms	164Dy	CD73	Surface	1.00
17	Ms	166Er	CD19	Surface	0.50
18	Ms	167Er	CD38	Surface	1.00
19	Ms	168Er	CD8a	Surface	0.25
20	Ms	169Tm	CD272 (BTLA)	Surface	1.00
21	Ms	170Er	CD161 (NK1.1)	Surface	0.50
22	Ms	171Yb	CD11b	Surface	0.25
23	Ms	172Yb	Fas	Surface	1.00
24	Ms	174Yb	CD223 (LAG-3)	Surface	1.00
25	Ms	176Yb	CD44	Surface	0.25
26	Ms	209Bi	CD11c	Surface	1.00
27	Ms	144Nd	Tcf1	Intracellular/Nuclear	0.50
28	Ms	146Nd	Gata3	Intracellular/Nuclear	1.00
29	Ms	158Gd	FoxP3	Intracellular/Nuclear	1.00
30	Ms	148Nd	ROR gamma (t)	Intracellular/Nuclear	1.00
31	Ms	154Sm	BATF	Intracellular/Nuclear	0.50
32	Ms	161Dy	Ki-67	Intracellular/Nuclear	1.00
33	Ms	175Lu	iNos (NOS2)	Intracellular/Nuclear	1.00
34	Ms	142Nd	Eomes	Intracellular/Nuclear	1.00
35	Ms	150Nd	IRF4	Intracellular/Nuclear	1.00

Supplemental Table 1. *Antibody panel used for CyTOF analysis.*
